# Perspectives of Nonphysician Clinical Students and Medical Lecturers on Tablet-Based Health Care Practice Support for Medical Education in Zambia, Africa: Qualitative Study

**DOI:** 10.2196/12637

**Published:** 2019-01-15

**Authors:** Sandra Barteit, Florian Neuhann, Till Bärnighausen, Annel Bowa, Sigrid Lüders, Gregory Malunga, Geoffrey Chileshe, Clemence Marimo, Albrecht Jahn

**Affiliations:** 1 Heidelberg Institute of Global Health Heidelberg Germany; 2 Harvard TH Chan School of Public Health Department of Global Health and Population Boston, MA United States; 3 Africa Health Research Institute (AHRI) KwaZulu-Natal South Africa; 4 Chainama College of Health Sciences Lusaka Zambia; 5 SolidarMed Lusaka Zambia; 6 School of Medicine University of Zambia Lusaka Zambia

**Keywords:** educational technology, computers, handheld, computer-assisted decision making, mobile apps, information dissemination, education, allied health occupations, Africa, Southern, Zambia

## Abstract

**Background:**

Zambia is faced with a severe shortage of health workers and challenges in national health financing. This burdens the medical licentiate practitioner (MLP) program for training nonphysician clinical students in Zambia because of the shortage of qualified medical lecturers and learning resources at training sites. To address this shortage and strengthen the MLP program, a self-directed electronic health (eHealth) platform was introduced, comprising technology-supported learning (e-learning) for medical education and support for health care practice. MLP students were provided with tablets that were preloaded with content for offline access.

**Objective:**

This study aimed to explore MLP students’ and medical lecturers’ perceptions of the self-directed eHealth platform with an offline-based tablet as a training and health care practice support tool during the first year of full implementation.

**Methods:**

We conducted in-depth qualitative interviews with 8 MLP students and 5 lecturers and 2 focus group discussions with 16 students to gain insights on perceptions of the usefulness, ease of use, and adequacy of self-directed e-learning and health care practice support accessible through the offline-based tablet. Participants were purposively sampled. Verbatim transcripts were analyzed following hypothesis coding.

**Results:**

The eHealth platform (e-platform), comprising e-learning for medical education and health care practice support, was positively received by students and medical lecturers and was seen as a step toward modernizing the MLP program. Tablets enabled equal access to offline learning contents, thus bridging the gap of slow or no internet connections. The study results indicated that the e-platform appears adequate to strengthen medical education within this low-resource setting. However, student self-reported usage was low, and medical lecturer usage was even lower. One stated reason was the lack of training in tablet usage and another was the quality of the tablets. The mediocre quality and quantity of most e-learning contents were perceived as a primary concern as materials were reported to be outdated, missing multimedia features, and addressing only part of the curriculum. Medical lecturers were noted to have little commitment to updating or creating new learning materials. Suggestions for improving the e-platform were given.

**Conclusions:**

To address identified major challenges, we plan to (1) introduce half-day training sessions at the beginning of each study year to better prepare users for tablet usage, (2) further update and expand e-learning content by fostering collaborations with MLP program stakeholders and nominating an e-platform coordinator, (3) set up an e-platform steering committee including medical lecturers, (4) incorporate e-learning and e-based health care practice support across the curriculum, as well as (5) implement processes to promote user-generated content. With these measures, we aim to sustainably strengthen the MLP program by implementing the tablet-based e-platform as a serious learning technology for medical education and health care practice support.

## Introduction

### Background

The severe shortage of health workers in sub-Saharan Africa is projected to worsen until 2030 and is aggravated by significant population growth [1]. This shortage is profoundly burdening the Zambian health care system. Although Zambia launched efforts to reduce this shortage [2], remote and rural areas are still severely underserved with only 7 clinicians per 10,000 people (urban areas: 16/10,000 people) [2-4] and insufficient coverage of health facilities [5]. Health workers are essential for closing the gap in delivering essential health services [3] and for moving forward toward universal health coverage and meeting health-related objectives of the sustainable development goals [6]. The Zambian National Health Strategic Plan for 2017-2021 [7] aims “to provide equitable access to cost-effective, quality health services as close to the family as possible” and specifically recognizes the need to “increase the proportion of rural households living within 5 km of the nearest health facility from the current 50% to 80% in 2030” [7]. Furthermore, efforts have been made to improve the allocation of current health workers throughout Zambia’s districts [2]. However, Zambia’s medical training institutions do not currently have the capacity to train the necessary number of health workers to sufficiently staff underserved areas [8].

In 2002, to alleviate the severe health worker shortage, the Ministry of Health initiated a medical licentiate practitioner (MLP) program at the Chainama College of Health Sciences (CCHS), Lusaka [9]. MLPs are nonphysician clinicians who receive shorter, skills-oriented medical training compared with physicians but can perform many traditionally physician-designated diagnostic and therapeutic tasks [10]. At first, the MLP program was implemented as a 2-year upgrade training for the cadre of clinical officers [4,11] and later transformed from a diploma-level degree to a Bachelor of Science degree in Clinical Sciences in 2013 [9]. A 1-year bridging course allowed diploma-holding MLPs to also acquire a Bachelor of Science degree at CCHS.

The importance of MLPs for Zambia’s health system has been shown, particularly in rural areas at the district level where the most critical shortages exist [9,10,12]. MLPs are trained in 4 main specialties: pediatrics, surgery, internal medicine, and obstetrics and gynecology. The last 2 years of the MLP training are focused on practical skills, whereby MLP students rotate every 8 weeks across health facilities, each with a medical focus in 1 of the 4 main specialties.

### Objectives

To strengthen the MLP program and specifically address the lack of medical lecturers and learning resources in practicum sites, a self-directed electronic health (eHealth) [13] platform (e-platform) that comprised technology-supported learning (e-learning) for medical education and health care practice support was introduced in 2016 as part of a more extensive blended learning approach [14]. The e-learning provides Web-based and offline access to static and interactive medical e-learning materials assembled and developed according to the existing curriculum, such as lecture notes, medical books, virtual patient cases, medical pictures, and videos on medical procedures. The health care practice support component provides access to standard treatment guidelines and medical algorithms to diagnose and treat patients. E-platform materials were generally pre-existing and not explicitly developed as electronically based materials. As training in the third and fourth study years includes clinical rotations in remote areas throughout Zambia, tablets were distributed (7 inches, Android-based) to all third- and fourth-year and bridging students to enable ubiquitous access to tablet-based content. The e-platform was implemented for Web access with the open-source Moodle software (Moodle HQ, Moodle Community), and MLP students were able to access contents with provided tablets or their own mobile devices. The content was downloaded to the tablets for offline usage via the Moodle mobile app; no internet connection was necessary to access the tablet’s contents, even in rural areas.

A pilot phase from January 2016 to August 2016 provided insight into shortcomings of the e-learning and eHealth platform (e-platform) that led to adaptations. The most significant change was an increase in available e-learning materials (including interactive learning materials), followed by a change in the tablet type that allowed mobile data access, which was highly requested by users. The first year of full implementation of the e-platform (September 2016 to August 2017) was evaluated qualitatively and quantitatively.

We present the results of the qualitative evaluation based on success factors for e-learning implementation as a framework to categorize themes. The objective was to explore students’ and medical lecturers’ perceptions of the self-directed e-platform as a tool for medical learning and teaching within the MLP program in the low-resource setting of Zambia to adapt and further develop the e-platform.

## Methods

### Research Context

A qualitative study design was used to explore the perceptions of MLP students and medical lecturers and evaluate the success of the implementation of the offline-based, self-directed e-platform in strengthening medical education in Zambia. The development of the evaluation framework was based on a prior pilot evaluation study of the MLP e-platform. The research methodology underpinning this study was content analysis [15]. Pilot evaluation results had identified shortcomings of the blended learning approach that needed improvement and are reported elsewhere [14]. In-depth interviews (IDIs) and focus group discussions (FGDs) allowed participants to state their opinions and experiences without the restrictions of predefined categories or terms. Bhuasiri et al’s research framework [16] provided the structure to categorize the identified themes within dimensions of a successful implementation of e-learning and particularly in low-resource settings (see Multimedia Appendix 1). We believed this framework was appropriate for the health care practice support component as health care practice support is also part of MLP education. With Delphi and analytic hierarchy process methods, Bhuasiri et al [16] identified the most relevant factors for e-learning success in low-resource settings and incorporated social cognitive and motivational theory, critical success factors, DeLone and McLean information system success, and the technology acceptance model. This framework was further adapted to reflect CCHS’s local setting and include factors that emerged during project implementation and this study’s data analysis. Bhuasiri et al’s research framework [16] includes 3 main dimensions of a successful e-learning implementation: personal, environmental, and system-related. The research framework unfolds into 7 subdimensions (learners and instructors’ characteristics, extrinsic motivation, e-learning environment, infrastructure, system quality, course and information quality, and institution and service quality). We further adapted and expanded these subdivisions (see Table 1) to incorporate the tablet-based e-platform in the research framework. Mobile learning in this context describes access to the MLP e-learning as well as health care practice support materials offline on mobile devices.

### Data Collection

We conducted 2 FGDs with MLP students, approximately 80 min each (see Multimedia Appendix 2 for the 17-item interview guide), and 13 IDIs with students and medical lecturers, approximately 20 to 40 min each (see Multimedia Appendix 3 for the 19-item discussion guide). In the IDIs, the e-platform is referred to as the e-learning platform. Interview guides for both the IDIs and FGDs complied with the questionnaire’s development guide for education research by Artino et al [17] and were based on a concise literature review, peer-based feedback rounds, and pilot testing. Moreover, 5 of the IDIs were conducted with medical lecturers and 8 with students. To reflect the study population, respondents were also purposively selected to proportionally represent gender and age groups. For each FGD, more students (n=10) than estimated necessary (n=6) were invited to ensure sufficient turnout to produce relevant information [18]. Age was categorized in 3 age groups: ≤35, >35 and ≤45, and >45 years (see Table 2 for details).

The group of medical lecturers was purposively drawn from the group of active lecturers in the third, fourth, and bridging study years within the MLP program. The IDIs with students were held in a secluded office within the MLP administration block on the CCHS campus and were conducted by the principal researcher, the first author of this manuscript, (German, female, doctoral student) in English, who was trained in information technology and global health and with over 10 years of experience in the field. The principal researcher engaged with study participants over short periods during project visits before the study’s commencement as part of the intervention implementation within the overall blended learning project. The 2 FGDs were conducted with students in lecture halls on the CCHS campus. The IDIs with medical lecturers were primarily conducted via phone (n=4) and 1 was held at the University Teaching Hospital in Lusaka. IDIs with medical lecturers and FGDs with students were guided by a facilitator (Zambian, female, master degree-level in Social Sciences) in English. The facilitator had no engagement with study participants before the study’s commencement.

At the time of the study, all students and medical lecturers were actively engaged in the MLP program. Furthermore, 2 student FGDs were considered sufficient to elicit the majority of prevalent themes [19]. Data saturation for IDIs was assumed according to the 10+3 criterion [20]. Field notes were taken during the interviews, and IDIs and FGDs were audio-recorded. During the IDIs and FGDs, questions were asked according to the interview and discussion protocol, thus prompting interviewees to provide further details until each line of inquiry was sufficiently covered.

**Figure 1:**
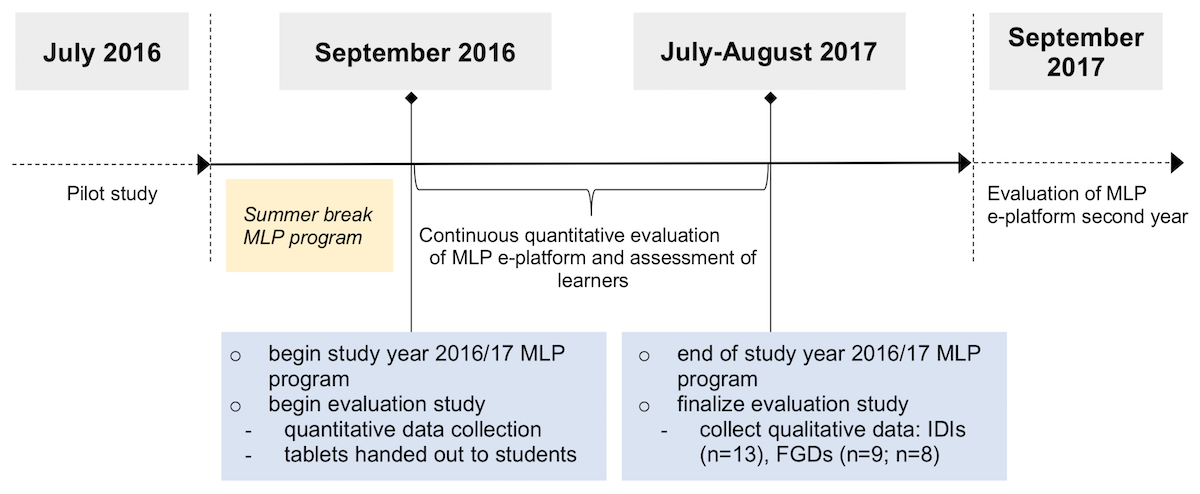
Timeline of study events in chronological order. MLP: medical licentiate practitioner; e-platform: e-learning platform with an eHealth component; IDIs: in-depth interviews; FGDs: focus group discussions.

The study began with the distribution of the tablets and the quantitative data collection in September 2016, after which IDIs and FGDs were held at the CCHS campus in July to August 2017 (end of the study year); therefore, the maximum exposure to the use of tablets was 12 months (see Figure 1, timeline of study).

**Table 1:** Bhuasiri et al’s research framework [16] for successful technology-supported learning (e-learning) implementation, with our additions for the tablet-based e-platform (marked with [add] in the table). The research framework has 3 major themes (individual dimension, environmental dimension, and system dimension) that unfold into subdimensions.

Dimensions			Term definitions
**1. Individual dimension**			
	**1.1 Learner’s characteristics**		
		Attitude toward tablet-based e-platform^a^	“Learners’ impression of participating in [m-learning^b^ or mHealth^c^] activities through [tablet] usage” [16]
		Focus on interaction	“The degree of contact and educational exchange among learners and between learners and instructors” [16] from the student’s perspective
	**1.2 Instructor’s characteristics (medical lecturers)**		
		Attitude toward tablet-based e-platform	Instructor’s “impression of participating in [m-learning/mHealth] activities through [tablet] usage” [16]
		Interaction fairness	“The extent to which the learner feels having been treated fairly regarding his or her interaction with the instructor throughout the [m-learning/mHealth] process” [16]
		Focus on interaction	“The degree of contact and educational exchange [...] between learners and instructors” [16] from the instructor’s perspective
	**1.3 Extrinsic motivation**		
		Perceived usefulness	“The degree to which a person believes that using [an m-learning/mHealth] system would enhance his or her learning performance” [16]
		Technological flexibility	The degree of flexibility that the technology is providing to users in a given setting [add]
		Expandability	The degree to which the provided m-learning and mHealth system and technology can be expanded according to user needs [add]
		Saving resources	The degree to which the provided m-learning and mHealth system and technology are saving users’ resources as measured by monetary spending, time, and additional characteristics [add]
		Punishment/restriction	The degree to which the provided m-learning and mHealth system and technology is restricting or punishing the user
**2. Environmental dimension**			
	2.1 Interaction opportunities		“Learner’s perceived interactions with others” [16] through m-learning and mHealth
**3. System dimension**			
	**3.1 Infrastructure and system quality**		
		Ease of use	“Refers to the degree to which the prospective user expects the use of [m-learning/mHealth] to be free of effort” [16]
		System functionality	“The perceived ability of [m-learning/mHealth] to provide flexible access to instructional and assessment media” [16]
		Technological adequacy	Refers to the degree to which the user expects the provided device to fit the setting and area of use [add]
		Technological quality	The quality of the provided device as measured by battery runtime, hardware reliability, operating system quality, and other characteristics [add]
		Internet quality	“The quality of the internet that can be measured by transmission rate, error rates, and other characteristics” [16]
	**3.2 Course and information quality**		
		Reliability	“Concerned with the degree of accuracy, dependability, and consistency of the information” [16]
		Relevant content	“The degree of congruence between what the learner wants or requires and what is provided by the information, course content, and services” [16]
	**3.3 Institution and service quality**		
		Sustainability of the e-platform	The degree to which m-learning and mHealth is implemented sustainably within the educational infrastructure [add]
		Tablet and e-platform training	“The amount of specialized instruction and practice that is afforded to the learner to increase the learner’s proficiency in utilizing [m-learning/mHealth] [...].” [16]
		Service quality	The quality of the service provided for m-learning and mHealth and the provided device

^a^e-platform: e-learning platform with an electronic health component.

^b^m-learning: mobile learning (with tablets and other mobile devices).

^c^mHealth: mobile health.

**Table 2:** Study participants of in-depth interviews and focus group discussions according to age groups and gender.

Age group (in years)	MLP^a^ students				Medical lecturers’ IDIs^b^	
	Female		Male		Female	Male
	IDIs	FGDs^c^	IDIs	FGDs		
≤35	2	3	0	6	0	0
>35 and ≤45	1	1	3	2	0	4
>45	1	1	1	1	0	1

^a^MLP: medical licentiate practitioner.

^b^IDIs: in-depth interviews.

^c^FGDs: focus group discussions.

### Data Analysis

The principal researcher recorded and then transcribed the students’ IDIs. Responses from all IDIs were coded by the principal researcher in 2 coding cycles to determine initial concepts with a commercial, qualitative data analysis program (NVivo, QSR International). Hypothesis coding was applied “to assess the researcher-generated hypothesis” [21]. The adapted research framework of Bhuasiri et al [16] directed the “researcher-generated, predetermined list of codes to qualitative data specifically to assess a researcher-generated hypothesis” [21], and the themes were, therefore, defined in advance (see the coding tree in Table 1, thematic areas from 1.1-3.3). The students’ and medical lecturers’ responses in the transcripts were coded according to the concepts and subsequent codes from Bhuasiri’s adapted research framework [21]. Coded elements were exported to a word processor where they were assigned to 1 theme. The principal researcher decided upon the most representative quotations to reflect the respective themes.

The FGD facilitator recorded and transcribed the student FGDs and medical lecturer IDIs. The principal researcher coded data for each participant in the student FGDs in sequence. The data of a randomly selected participant were coded first and only then were the data of another randomly selected participant coded. The hypothesis coding included 3 major dimensions (or themes): individual, environmental, and system. A total of 7 categories emerged in the students’ and medical lecturers’ responses: (1) learner’s characteristics, (2) instructor’s characteristics, (3) extrinsic motivation, (4) interaction opportunities, (5) infrastructure and system quality, (6) course and information quality, and (7) institution and service quality.

Transcripts were not returned to participants for comments or corrections as it was not feasible in the given setting. However, the interview answers were validated directly after the IDIs as interview answers were run through question-by-question and answer-by-answer with each interviewee. Furthermore, IDI responses were validated with responses from the FGDs. All data were pseudonymized before data analysis. This study adhered to the consolidated criteria for reporting qualitative research checklist [22].

### Ethical Considerations

The study protocol was approved by the Biomedical Research Ethics Committee of the University of Zambia and the ethical committee of the University Hospital Heidelberg, Germany. Before taking part in this study, all participating students and medical lecturers agreed to an informed consent that explained the scope and purpose of this study and the right to withdraw at any point. All participants gave written consent and were treated with respect.

## Results

### In-Depth Interview Characteristics

Out of a total of 81 MLP students and 23 medical lecturers, 8 MLP students (female n=4 and male n=4) and 5 medical lecturers (male n=5) had IDIs. IDIs lasted from 26 to 47 min, with an average of 33 min. Out of 10 invited participants per student FGD, the first FGD included 3 female and 6 male students from the third study year (duration: 78 min) and the second had 3 female and 5 male students from the fourth and bridging study years (duration: 88 min). Results are presented according to the respective coding themes of the hypothesis coding, and dimension themes are indicated in italics in the text that follows.

The interviewees are referred to in the text as follows: for IDIs, *interviewees* 1 to 8 are student participants and *lecturers* 1 to 5 are medical lecturers and for FGDs, *respondents* 1 to 9 are third study year students, for example, respondent 1 (third) and respondents 1 to 7 are fourth study year and bridging students, for example, respondent 1 (fourth/bridging).

### Individual Dimension

#### Learners’ Characteristics (Students)

The *attitude* of students *toward the tablet-based e-platform* was generally quite positive as interviewee 1 (third) expressed:

I think we were the lucky ones, it [tablet-based e-platform] came at the right time.

Some students requested more *focus on interaction* by lecturers regarding the integration of tablets in their lectures, as respondent 6 (third) stated:

We would have lectures, then she [lecturer] would like to post whatever she would teach or any new guidelines. She would make sure that it is made available to us on the e-learning platform. So, if the other lecturers follow suit and try and take her example.

#### Instructors’ Characteristics (Medical Lecturers)

The *attitude* of medical lecturers *toward the tablet-based e-platform* was also quite positive, as lecturer 1 said:

I think it’s a platform [that] ought to move forward, a platform that we ought to support.

In general, the e-platform was well perceived; however, some lecturers recognized themselves as nonusers or low-users, such as lecturer 4:

Yes [my] general view is it is a very good program, but I think it is still meeting challenges whereby we [medical lecturers] have very few accesses, may be due to our time

Lecturer 1, identified as a low-user who was only involved at one point in time, said:

Yes I have done it [uploading] two times. Well, because when I uploaded that material, I thought it was up-to-date, but I have not been visiting the site.

*Reasons for instructor low usage* and participation were explained by lecturer 1:

First of all, the lecturers need to have tablets; number two, they need to have connectivity, internet connectivity; number three, there needs to be a platform where lecturers and students and other lecturers interact and discuss this.

Lecturer 3 identified the need for:

...office space. If you are given an opportunity to go there and sit in the staff room or lecturers’ room, then that willhelp you to have more preparations and access to the platform.

Lecturer 2 attributed the low usage to the newness of the e-learning program and the need for face-to-face training:

I don’t think they [medical lecturers] are using it [tablet-based e-platform] actively; some are, some are not. I think it is a developing program. So, I think it is still in the early phases...Lecture notes, there will always be need to explain in person. The videos I think will never be enough, so there will always be a need actually to explain and explain and explain in person. You must actually be there in the ward and do things on the patient...

To increase the level of *interaction*, lecturer 3 suggested regular content review meetings and the introduction of e-based assignments:

...the only way we can improve on that is to have a regular review of the material that we have contributed...If assignments are scheduled at specific intervals, and both, the students and the lecturers are expected to either review or revise those assignments, probably that may stimulate the lecturers to use the platform much more regularly, including the students.

Lecturer 1 recognized that a question-and-answer section would be beneficial:

If I am given an opportunity to interact with students, I will be answering their questions or their concerns. Then, I will log in specifically for those questions and try to answer them...it should be a two-way thing.

#### Extrinsic Motivation

Many students *perceived* the tablet as *useful* for medical practice as a quick reference tool. They welcomed the *technological flexibility* for patient care, as respondent 7 (third) explained:

Basically, a tablet is, you will have everything. You have books, it is like everything is compressed into that, and it has been very useful, very useful with the patient you need to consult. There are treatment guidelines. If you are trying to calculate something, it is a very useful gadget.

Students reported that the tablet provided them with greater study flexibility, as interviewee 5 described:

Wherever I am, I’m walking on the road, I’m on the bus, I can easily open - then do studies. Now with that[tablet], it has encouraged me to study.

Interviewee 6 specified:

I get access where there’s internet or not. It’s [tablet] really contributing immensely.

Students appreciated tablet *expandability* also, as respondent 8 (third) highlighted:

Actually these tablets they also allow students to put [upload] their own books, so it is very useful.

A few students stated that the tablet *saved them money*, as interviewee 1 said:

I don’t have to pay so much for books anymore. I don’t have to always have internet bundles to access information when I need it.

A few students did not find the tablet useful in *saving time*, as interviewee 7 explained:

There are a lot of things. The period that I came as a student, I wasn’t treated as a student, I was treated as manpower. So, I didn’t have much time, besides for an exam, which I was focusing on...My fear is that I might waste time. So, I just concentrate on what is important at present for the sake of exams because my period is short.

The tablet was also perceived as a *restriction*, as interviewee 7 stated:

Sometimes I used to leave it [tablet] in the room, for security purposes. Especially if you lose the tablet, you’ll pay. So, I was extra careful. So, it was a bit risky, but sometimes I would go with it.

### Environmental Dimension

#### Interaction Opportunities

Some students requested an addition to the e-platform to foster *interaction*, for example, respondent 9 (third) said:

What I would like to see is student participation. Could there be like a forum where students can add questions and lecturers will respond with teaching materials based on what the student is requesting, or at least good feedback?Learning,it is student-based.

### System Dimension

#### Infrastructure and System Quality

In general, opinions on the *ease of use* varied among individuals. However, the majority of students seemed to use the tablets with ease, as respondent 7 (fourth/bridging) exemplified:

I only had challenges just a few weeks after we were given the tablets. I don’t have much challenge. I think certain challenges are being expressed maybe at the individual level.

Some students perceived the tablets as an additional burden, as respondent 3 (fourth/bridging) stated:

I don’t know why the tablets have to be made so complicated, such that we need training. Already we have got a lot of work, a lot of things to study. So, [it] is like we are taking another profession in IT, those things.

To enhance the ease of use for students, lecturer 2 suggested keeping the e-platform materials clear and concise:

I think to make it [e-platform] more user-friendly is [to] remember that the students do not have a lot of time. So, like anybody else, you want to go to a material,which is easily accessible, easily understood without a lot of homework. Welive in an age where there is too much information elsewhere. If I just read one lecture on the tablet and I don’t need to resort to a textbook, then I will go to that particular lecture. So, it’s a question of how user-friendly are thematerials on the tablets.

*System functionality* was perceived as challenging in regard to comprehensive learning materials, as interviewee 2 mentioned:

Because you’ll find that, you scroll, scroll, scroll, scroll.

To this end, interviewee 1 suggested:

most of the presentations will have to be converted to a tablet-acceptable version.

In general, the tablet as a learning and health care practice support technology was found to be *adequate* within the given setting, especially for the hospital setting, as respondent 5 (third) said:

with the coming of the e-learning tablets it is something that is easy to carry whenever you are faced with a challenge when you are maybe with a patient.

Many students identified the quality of the tablets as inadequate. The primary concern was the tablet battery, as respondent 2 (fourth/bridging) stated:

within a few hours my tablet drains. And sometimes even if you are not using it, it heats up and just after a few hours, the battery finishes.

Some students perceived the tablet as too unreliable for medical practice, as interviewee 4 explained:

When you need it [tablet], if the patient is in front of you, you need to clarify something, it [e-platform tablet application] fails to open, you get stuck.

Other concerns regarded repairs and spare parts, as a respondent (third) summarized:

When the battery finishes, we don’t know where to get these batteries. So, next time when they are buying these things they should at least consider things [tablets] that we can easily find here locally.

Respondent 4 (fourth/bridging) was concerned about:

what happens if three, four months from now the tablet may cease, is there anything that will happen or that’s the end of everything?

*Internet quality* was not perceived as crucial, as lecturer 3 stated:

It [e-platform] takes off the burden of the need for the students to look for internet access, especially in their remote areas where internet access is a challenge.

However, access to the internet was still perceived as necessary beyond what was available as materials on the tablets, as respondent 8 (fourth/bridging) highlighted:

The e-learning platform worked with the loaded materials. But now out of the academic situation, we need to go and explore so that with every situation that we may encounter, we may be able to go online.

#### Course and Information Quality

With regard to the subtopic of reliability, a majority of the students perceived the *reliability* of the available materials on the e-platform as inferior, as interviewee 7 expressed:

I was very disappointed with the tablets the time they gave us. Why I got so disappointed is some of the notes, which they had put in the tablet, they were old notes. Some of them as early or as late as 2005. Medicine is dynamic. It keeps on changing. So, this is 2017. If you put notes for 2005, 2006 it’s not fair enough for the student.

Interviewee 6 also highlighted the need for updates but was more content with the currently available learning materials:

Currently, I think it’s enough. So, sometimes I feel maybe we are reading what is not latest and out of the bulletin medical journals.

As a way to increase the quality and timeliness of the learning materials, lecturer 5 suggested the following:

have at least two [review meetings] in a quarter so that our contacts with the coordinators of the program and the Chainama [CCHS] staff also is increased, having more interaction and all viewpoints shared.

Despite the identified shortcomings in reliability, many students found the information that was available on the e-platform to be generally *relevant* to their studies and medical practice. Interviewee 5 said that:

...especially when we are on the ward, we are doing our clinical work, it’s easy to access information from the e-learning platform. And, most of the things that we were doing and what we are doing is actually there. It has made my life easier, in that I’m able to access the information I need without moving with the laptop, everywhere I go.

Respondent 2 (fourth) revealed that he mainly engaged with the e-platform for exam preparations:

But then, of late just before the exams, that’s when I could hear my friends say, they could tell me there is this there, that a lot of material that’s when I got interested.

Although the majority of students identified a lack of practical materials and requested additional e-learning materials, for example, respondent 9 (third) stated:

It lacks the practical information. So, I feel we need to add more lecture notes, which should come from the lecturers who are actually teaching us from here. They should put something that will help the student to know how to answer the examination questions. Some people are doing these OSCE exams for the first time.

Respondent 1 (third) felt that she did not find relevant content:

...I just use it as a skeleton. It doesn’t have enough meat that I need. So, we really need to put a lot of data in the Moodle [e-platform] for us to be able to use them effectively.

Respondent 5 (fourth/bridging) remarked that it would be beneficial:

if I were [in a rural health facility], you find that certain equipment is not there, but I need to save a life, so I should also be taught alternatives when I am faced with such situations.

#### Institution and Service Quality

To reach a *sustainable* *e-platform* at CCHS, lecturer 3 suggested involving the Ministry of Health and a fee-based system:

...to sell the idea [e-platform] with strong advocacy to the training directorate at the Ministry of Health. By doing so probably it gives Chainama [CCHS] as a college much more privilege when it comes to asking for extra funding. I am not sure in terms of how much students contribute towards the tablets,...just a small percentage to contribute towards the acquisition of replacement tablets.

Speaking in regard to operationalization, lecturer 2 emphasized:

The ownership should sit in the college, especially in the department of medical licentiate training. That’s where the ownership should be, that’s the coordinating center, that’s the connecting center for both those that teach and the students that pass through the program. Somebody should be identified in the department who can be the main person coordinating the e-learning platform because a lot of reviews, a lot of materials need to be developed over time.

*Training for the tablet-based e-platform* was clearly stated as a necessity by the majority of students and medical lecturers as respondent 5 (third) mentioned:

I have had this tablet for some time. I don’t know anything on how to access it because I would log in and try to click somewhere and it will ask for a password, and I don’t even know how to press that. There is a need for training.

More in-depth training from information technology (IT) support was also requested by respondent 3 (third):

I think at some point it would be right if we had people from the IT to come and just explain to us to say that okay when you see it behaving like this, the best way you are supposed to do is A-B-C. In that way, we are able to understand how to manage and operate the gadget nicely.

Training formats were suggested as a 1-day class-based session with a concluding test or as individual training sessions with trained student experts for the e-platform, as interviewee 5 proposed:

Certain individuals might not have the computer literacy orexperience.But for those, I think, maybe it can be better and cost-effective if you can arrange with individuals. They can easily contact their colleagues who know how to do it. They can be taught, yes. Better to go to friends they’re close to.

A tutorial was also suggested by respondent 6 (fourth/bridging):

...maybe we come up with some form of a booklet where you put those steps that someone needs to follow, to open this and that, I think it would help.

Medical lecturers suggested a training session incorporating a content revision, for example, lecturer 1 proposed:

I think a refresher. So that people are reminded of uploading materials.

Lecturer 5 proposed the training:

as a review process also, to get feedback from them [medical lecturers] on how it [e-platform] is working and how best it can be made use of.

The *service quality* provided by the CCHS IT staff was perceived as helpful and accessible as respondent 3 (third) stated:

I think the IT department has been very helpful...For me personally, every time that it [tablet] had malfunctioned, they try all their best to fix it.

Students welcomed the available support, as respondent 7 (third) mentioned:

When you are using electronic gadgets, there are always few challenges. After upgrading it [tablet] stopped functioning, and we didn’t know how to repair them. Until the people from the IT came.

One concern regarded IT support after graduation, as respondent 9 (third) remarked:

Right now, when the tablet is not working, there is an internet problem the IT guys are available. Or at least we’ve got access channels. Probably in the next coming years, there will still be problems. So how do we access them [IT] once we are no longer here?

## Discussion

### Principal Findings

Participants in FGDs and IDIs offered a range of insights into their perceptions of the tablet-based e-platform that comprised e-learning for medical education with an eHealth component. Overall, the perceptions were positive and varied toward the tablet-based e-platform as a tool for medical education and health care practice support at CCHS. The results of the evaluation proved useful for the further development of the e-platform as students’ and instructors’ perceptions of the needs and shortcomings of the tablets and the e-platform were identified.

A significant advantage was seen in the tablet-based offline component as it only required an internet connection for content updates. The tablet was adequate for the setting (especially for medical practice) as it fitted into the doctors’ coat pockets and served as a handy reference. Quality weaknesses of the tablet were identified as fragility, fast-draining battery, and *buggy* operating system that frequently crashed or froze. The specific tablet model had been chosen as the best quality at an affordable price. Although it would be beneficial to use tablets available on the Zambian market, long-term tablet support will remain a challenge. Changes in technology and tablet manufacturer priorities require a continued investment to keep pace with changing technologies.

Some students believed they were missing curriculum content and tablet functionality. A few were overwhelmed by the tablet and perceived it as an extra burden to an already demanding study schedule. Comprehensive half-day training sessions at the beginning of the study year may enable productive usage of the tablet and the e-platform.

Both students and medical lecturers agreed that updated learning materials was one of the most significant needs, although the overall view on available learning materials of the e-platform was generally positive. The learning materials available at the time of the evaluation were gathered from various sources but were not optimized or designed as e-learning materials. Providing materials that follow multimedia principles could be beneficial as they were shown [23] to provide better learning outcomes and potentially improve the quality of the e-learning platform and its eHealth component. Incorporating teaching methods that foster learning from surface to long-term memory, overlearning strategies such as practice tests, giving and receiving feedback, and spacing practice over time may all be beneficial [24]. Addressing student evaluations may increase self-evaluation and empower students to become more independent learners [24]. However, the limited number of lecturers poses a bottleneck. To this end, student engagement in creating content, so-called user-generated content, may be a potential solution [25]. Students could employ available mobile devices to generate content such as short videos on medical procedures. If, when preparing a topic for a short video, students read through the current medical guidelines and prepare the topic as an e-learning multimedia content, then they will also practice their pedagogical skills. Hence, user-generated content fosters medical learning and creates much-needed content for the e-platform.

Although lecturers were positive about the e-platform, they stated they were low-users or nonusers who rarely made use of the e-platform. Thus, their expressed attitude does not seem to be a reliable indicator of their actual involvement and usage. Moreover, their answers may have been biased by social desirability [26] and an expected openness to technologies. However, their overall positive answers may indicate a certain willingness and preparedness for more active, future involvement in the e-platform, and medical lecturer engagement is pivotal for updating and creating learning materials. As the numbers of medical lecturers are unlikely to substantially increase in the next few years [27,28], an e-platform coordinator could be key for the MLP program [29]. Regular lecturer review meetings could potentially foster content updating and the creation of new learning materials and also strengthen lecturer ownership of the content and pedagogy of learning materials [30]. Changes introduced with new learning technology should be constructively discussed, and barriers for implementation should be identified. Such operational structures may promote and support a sustainable e-platform.

Furthermore, we want to employ design thinking methods in the ongoing evaluation framework [25] to explore user habits and better understand the user and their challenges and increase adoption of digital technologies, especially by medical lecturers. As a next step for the MLP e-platform, e-learning modules are to be developed for all respective mandatory curriculum items. The construction of the initial e-learning modules is a substantial one-time effort mainly involving medical lecturers and IT. The quality and quantity of course modules on the e-platform and their respective content benefit from regular reviews and corresponding adjustments. Medical lecturers potentially may be more mindful of these e-platform tasks if these were included in the medical teaching schedule and teaching responsibilities, as well as if these topics were part of their continuous training and assessment. To empower medical lecturers to actively teach with the e-platform, we consider powerful training contents to comprise learning about engaging teaching strategies on how to best employ e-learning in a blended learning setting for medical education, as well as more technical skills like to work with the learning management system. Continuous costs for the MLP e-platform include training of involved staff, licensing costs of learning materials, authoring software for content and course development, as well as running and maintenance costs of IT infrastructure.

E-learning promotes a shift during which lecturers “become facilitators of learning and assessors of competency” instead of “distributors of content” [31]. This shift should be reflected in the curriculum [32]. To strengthen the e-learning infrastructure at CCHS, training for all involved need to be increased, which could be realized as weekly meetings with IT technicians to support a community of practice [33]. In the future, training for medical lecturers could be implemented as small learning units, so-called micro-learning sessions [34], which include short instructional videos on the e-platform and which can be more easily integrated into the clinical workday. Other methods to increase adoption may constitute so-called e-platform champions [35,36] who become points of reference as they are trained more intensely in e-learning and eHealth methodologies. Currently, the IT offices are separated from the administrative and medical lecturers’ offices. Moving the IT staff physically closer to the MLP program administration’s offices could increase their involvement in the educational processes of the e-platform and strengthen their role beyond simple IT support [33]. E-learning and eHealth can provide fertile learning environments that strengthen medical education and quality of care, but they need to be embedded in the curriculum with recognition of their strengths and shortcomings.

### Limitations

The study has several limitations. First, as there are infrastructural restrictions on the total number of students per year, the study population based on the general MLP student population comprises only a small number of students. Second, we cannot rule out that the study participants’ behavior would have differed if the researcher and discussion assistants had not been present. We tried to minimize this limitation by involving an outside person to conduct the FGDs and IDIs with the medical lecturers, in addition to the principal researcher who conducted the student IDIs. Third, it was not possible in the framework of this study to have study participants verify interview transcripts. We attempted to minimize this limitation by having the researchers review each question and respective respondent’s answer at the end of each interview to verify the accuracy of the respondents’ statements. Fourth, FGDs in this evaluation period could only be conducted with students, potentially diminishing the insight from the medical lecturers. Fifth, the study employs Bhuasiri’s [16] framework, which potentially does not account for all involved dimensions and characteristics of a technology-enhanced intervention for medical education, and thus, further research to refine, adapt, and develop this framework accordingly may be required. Sixth, this study took place in the Zambian MLP program, so there are limitations on how far findings can be generalized to other clinical settings.

### Conclusions

A self-directed, tablet-based e-platform that comprised e-learning with an eHealth component was introduced and proved feasible in the low-resource setting of Zambia. The qualitative evaluation included FGDs and IDIs that identified shortcomings, limiting the broad adoption of the e-platform among students and medical lecturers. To improve on the shortcomings of the e-platform in this setting, comprehensive half-day training sessions at the beginning of the study year may increase the ease of use with provided tablets for students and medical lecturers. User-generated content, the nomination of an e-platform coordinator and an e-platform steering committee, as well as an e-platform embedded in the curriculum could improve the quality and quantity of learning content that has been perceived by students as outdated and insufficient. Furthermore, supporting regular meetings for medical lecturers to review and discuss the contents of the e-platform may increase engagement for technology-enhanced learning and teaching as well as use as a health care practice support tool. Institutional changes such as moving the IT department physically closer to the MLP program’s medical lecturers and administration may foster vital exchanges that increase understanding and adoption of e-learning for medical education.

The implementation of digital learning environments such as e-learning and eHealth is a multidimensional process that ideally is cyclically iterated [25] to identify and understand the users’ needs and actuators. A clear objective and a serious commitment are required within the implementing institution, and the effort of all involved is necessary to sustainably implement an e-platform as a beneficial learning and teaching method and a health care practice support tool for students and medical lecturers.

## Appendix

Multimedia Appendix 1 Table of Bhuasiri et al’s research framework with 3 main dimensions and subdimensions adapted to the setting of the medical licentiate program.

Multimedia Appendix 2 Interview guide: in-depth interviews.

Multimedia Appendix 3 Interview guide: focus group discussions.

## Abbreviations

CCHS: Chainama College of Health Sciences
eHealth: electronic health
e-learning: technology-supported learning
e-platform: e-learning platform with an eHealth component
FGD: focus group discussion
IDIs: in-depth interviews
IT: information technology
MLP: medical licentiate practitioner
