# Authorship Correction: Perspectives of Nonphysician Clinical Students and Medical Lecturers on Tablet-Based Health Care Practice Support for Medical Education in Zambia, Africa: Qualitative Study

**DOI:** 10.2196/13431

**Published:** 2019-04-03

**Authors:** Sandra Barteit, Florian Neuhann, Till Bärnighausen, Annel Bowa, Sigrid Lüders, Gregory Malunga, Geoffrey Chileshe, Clemence Marimo, Albrecht Jahn

**Affiliations:** 1 Heidelberg Institute of Global Health Heidelberg Germany; 2 Harvard TH Chan School of Public Health Department of Global Health and Population Boston, MA United States; 3 Africa Health Research Institute (AHRI) KwaZulu-Natal South Africa; 4 Chainama College of Health Sciences Lusaka Zambia; 5 SolidarMed Lusaka Zambia; 6 School of Medicine University of Zambia Lusaka Zambia

The authors of “Perspectives of Nonphysician Clinical Students and Medical Lecturers on Tablet-Based Health Care Practice Support for Medical Education in Zambia, Africa: Qualitative Study” (JMIR Mhealth Uhealth 2019;7(1):e12637) inadvertently omitted Annel Bowa, MSc (Chainama College of Health Sciences, Lusaka, Zambia) from the list of authors, although initially he had been included as a co-author and had signed the License to Publish form. He has now been added to the list of authors directly after Till Bärnighausen.

The correction will appear in the online version of the paper on the JMIR website on April 3, 2019, together with the publication of this correction notice. Because this was made after submission to PubMed, PubMed Central, and other full-text repositories, the corrected article also has been resubmitted to those repositories.

## Appendix

Multimedia Appendix 1 Letter from authors requesting correction.

