# Supplementary material for: Perspectives of Nonphysician Clinical Students and Medical Lecturers on Tablet-Based Health Care Practice Support for Medical Education in Zambia, Africa: Qualitative Study
Source: JMIR Mhealth Uhealth. 2019 Jan 15;7(1):e12637. doi: 10.2196/12637 (PMC6350089; doi:10.2196/12637)
Supplement: Multimedia Appendix 1 [file mhealth_v7i1e12637_app1.pdf]

**Multimedia Appendix 1. Table of Bhuasiri et al.'s research framework with three main dimensions and sub-dimensions adapted to the setting of the medical licentiate program.**

| **Individual Dimension** | **Term Definitions** |
|---|---|
| *Learner's characteristics (students)* | |
| - Attitude towards tablet-based e-platform | "Learners' impression of participating in [m-learning/mHealth] activities through [tablet] usage" [14] |
| - Focus on interaction | "The degree of contact and educational exchange among learners and between learners and instructors" [14] from the students' perspective |
| *Instructor's characteristics (medical lecturers)* | |
| - Attitude towards tablet-based e-platform | Instructors' "impression of participating in [m-learning/mHealth] activities through [tablet] usage" [14] |
| - Interaction fairness | "The extent to which the learner feels having been treated fairly regarding his or her interaction with the instructor throughout the [m-learning/mHealth] process" [14] |
| - Focus on interaction | "The degree of contact and educational exchange […] between learners and instructors" [14] |
| *Extrinsic motivation* | |
| - Perceived usefulness | "The degree to which a person believes that using [an m-learning/mHealth] system would enhance his or her learning performance" [14] |
| - Technological flexibility | The degree of flexibility that the technology is providing to users in a given setting |
| - Expandability | The degree to which the provided m-learning and mHealth system and technology can be expanded according to user needs |
| - Saving resources | The degree to which the provided m-learning and mHealth system and technology are saving users' resources as measured by monetary spending, time and additional characteristics |
| - Punishment/restriction | The degree to which the provided m-learning and mHealth system and technology is restricting or punishing the user |
| **Environmental Dimension** | |
| - Interaction opportunities | "Learner's perceived interactions with others" [14] through m-learning and mHealth |
| **System Dimension** | |
| *Infrastructure and system* | |

| | |
|---|---|
| *quality* | |
| - Ease of use | “Refers to the degree to which the prospective user expects the use of [m-learning/mHealth] to be free of effort” [14] |
| - System functionality | “The perceived ability of [m-learning/mHealth] to provide flexible access to instructional and assessment media” [14] |
| - Technological adequacy | Refers to the degree to which the user expects the provided device to fit the setting and area of use |
| - Technological quality | The quality of the provided device as measured by battery runtime, hardware reliability, operating system quality, and other characteristics |
| - Internet quality | “The quality of the internet that can be measured by transmission rate, error rates, and other characteristics” [14] |
| *Course and information quality* | |
| - Reliability | “Concerned with the degree of accuracy, dependability, and consistency of the information” [14] |
| - Relevant content | “The degree of congruence between what the learner wants or requires and what is provided by the information, course content, and services” [14] |
| *Institution and service quality* | |
| - Sustainability of the e-platform | The degree to which m-learning and mHealth is implemented sustainably within the educational infrastructure |
| - Tablet and e-platform training | “The amount of specialized instruction and practice that is afforded to the learner to increase the learner’s proficiency in utilizing [m-learning/mHealth][…]” [14] |
| - Service quality | The quality of the service provided for m-learning and mHealth and the provided device |
