# Supplementary material for: Perspectives of Nonphysician Clinical Students and Medical Lecturers on Tablet-Based Health Care Practice Support for Medical Education in Zambia, Africa: Qualitative Study
Source: JMIR Mhealth Uhealth. 2019 Jan 15;7(1):e12637. doi: 10.2196/12637 (PMC6350089; doi:10.2196/12637)
Supplement: Multimedia Appendix 2 [file mhealth_v7i1e12637_app2.pdf]

Multimedia Appendix 2. Interview guide: in-depth interviews.

Semi-structured interview guide – ML Lecturers

Name:	First Name:

**Name of interviewer**	
**Time at start of interview (hh:mm, 24h)**	
**Time at end of interview (hh:mm, 24h)**	
**Date of interview**	
**Place of interview**	

Introduction (Reference to ICF)

1. Introduce yourself.
2. Explain purpose of interview.
3. Address terms of confidentiality.
4. Explain format of the interview.
5. Indicate how long interview usually takes.
6. Tell interviewee how to get in touch with you later if interviewee wants to.
7. Ask interviewee if there are any questions before interview starts.

Guiding Questions

1. What are your views regarding the e-learning platform for the ML program?

2. What do you think about tablets used as learning devices for the ML program?

3. Do you think lecturers are using actively the ML e-learning platform? Why?

4. Do you think students are using actively the ML e-learning platform? Why?

5. What do you think could be e-learning materials that are most useful for the ML e-learning (regarding your specialty)?

6. How would you keep the quality of the materials on the ML e-learning platform high? How could the ownership of the ML e-learning platform be handed over to ML lecturers and site consultants?

7. Would you be comfortable with a content checklist for your lecture slides? Would you be comfortable with a student enriching your lecture slides with multimedia content (x-ray pictures, smears, microscope, audio of heart rhythm or breathing, or videos of medical procedures) and additional questions (to make use of all features of the e-learning platform)?

8. What do you think are the biggest challenges of an e-learning platform for the ML program? How could they be overcome?

9. How do you think we could integrate you as lecturer to provide materials for the ML e-learning platform frequently (and regular material updates)?

10. How do you usually prepare for your lectures for the ML programme?

11. What is your preferred method of teaching and training? From your perspective, which teaching and training method is most effective with ML students?
    - ⇒ Why?

12. Do you usually use technology for your lectures? *How often? What ways have you used technology and the web for lecturing within the ML programme*?
    - ⇒ If yes, why?
    - ⇒ If no, why?

13. What factors encourage you to use technology in teaching?

14. What factors discourage you to use technology in teaching?

15. How do you see yourself using the e-learning/m-learning? From your perspective, how will you make use of it?
    - ⇒ What kind of training could help you make better use of e-learning/m-learning?

16. How do you think the e-learning/m-learning could be well integrated into everyday teaching? How do you think the involvement of the lecturers could be improved with the ML e-learning? (increasing the involvement of the lecturers?)

17. What are your recommendations for the ML e-learning (regarding your specialty)?

End of interview

1. Summarize key points of the respondent.
2. Ask the interviewee if there is anything they would like to add.
3. Thank interviewee for their time.

Semi-structured interview guide – ML Students

**Name of interviewer**	
**Time at start of interview (hh:mm, 24h)**	

**Time at end of interview (hh:mm, 24h)**	
**Date of interview**	
**Place of interview**	

Introduction

8. Introduce yourself.
9. Explain purpose of interview.
10. Address terms of confidentiality.
11. Explain format of the interview.
12. Indicate how long interview usually takes.
13. Tell interviewee how to get in touch with you later if interviewee wants to.
14. Ask interviewee if there are any questions before interview starts.

Guiding Questions

1. Why did you decide to train as a Medical Licentiate? What motivated you to join the ML training programme?

2. What do you expect from the ML training? What are your priorities in studying within the ML training programme?

3. Has the medical training met your expectations so far?
    If yes, why?
    If no, why?

4. What do you enjoy most in your training? Can you give an example? What, if any, positive aspects/experiences have you had regarding the ML training?
    Why?

5. What do you enjoy least in your training? Can you give an example? What, if any, negative aspects/experiences have you had regarding the ML training?
    Why?
    What is difficult?

6. What hurdles remain to you as a student of the ML training programme?

7. How do you get along with the e-learning/mobile learning? How do you wish the ML e-learning/m-learning would support you in regards to the ML training?

8. How do you use the tablet for medical educational purposes? How frequently? In which ways have you used e-learning and m-learning for your ML training? How have you employed the tablet/e-learning platform for your ML training? Could you describe a typical day/week?

9. How do you wish the lecturers would employ the e-learning/m-learning at CCHS?

10. What do you enjoy most about mobile learning with the ML tablet?

11. What do you enjoy least about mobile learning with the ML tablet?

12. How do you think the e-learning/m-learning could be improved for the ML programme? Where do you think the e-learning/m-learning is in 5 years?

End of interview

4. Summarize key points of the respondent.
5. Ask the interviewee if there is anything they would like to add.
6. Thank interviewee for their time.
