# Supplementary material for: Perspectives of Nonphysician Clinical Students and Medical Lecturers on Tablet-Based Health Care Practice Support for Medical Education in Zambia, Africa: Qualitative Study
Source: JMIR Mhealth Uhealth. 2019 Jan 15;7(1):e12637. doi: 10.2196/12637 (PMC6350089; doi:10.2196/12637)
Supplement: Multimedia Appendix 3 [file mhealth_v7i1e12637_app3.pdf]

Multimedia Appendix 3. Interview guide: focus group discussions

Focus Group Discussion – ML Students

Participants - Female:	Participants - Male:

**Name of focus group moderator**	
**Time at start of discussion (hh:mm, 24h)**	
**Time at end of discussion (hh:mm, 24h)**	
**Date of focus group discussion**	
**Place of focus group discussion**	

Introduction

1. Welcome and Introduce yourself.
2. Explain purpose of focus group discussion:
   The reason we are having this focus group discussion is to find out whether and how teaching and training for the ML training programme can be enhanced with the support of educational technology, also with respect to the skills and knowledge required for the daily work of ML practitioners.
   We need your input and want you to share your honest and open thoughts with us.
   You were selected because you are directly involved in the current ML training programme, such as e-learning.
3. Address terms of confidentiality (ground rules):
   a. I want you to do the talking.
      I would like everyone to participate. I may call on you if I haven't heard from you in a while.
   b. There are no right or wrong answers, only differing points of view.
      Every person's experiences and opinions are important.
      You do not need to agree with others, but you must listen respectfully as others share their views.
      Speak up whether you agree or disagree.
      We want to hear a wide range of opinions.
   c. I ask you to turn off your mobile phones. If you cannot and if you must respond to a call, please do so as quietly as possible and rejoin us as quickly as possible.
   d. I will be tape recording the discussion, because I want to capture everything you have to say.
      I do not identify anyone by name in the report.
      You will remain anonymous.
   e. What is said in this room stays here.
      I want you to feel comfortable sharing when sensitive issues come up.

4. Explain format of the focus group discussion.
5. Indicate how long discussion usually takes.
6. Tell participants how to get in touch with you later if participants wants to.
7. Ask participants if there are any questions before interview starts.

Guiding Questions

Students understanding, experiences and views of the ML e-learning platform

1. What do you understand under the ML e-learning platform?
   - For how long has the ML e-learning platform been in existence?
   - Were you given an orientation on how to use the e-learning platform and the tablet?

2. What are your views on the ML e-learning platform?

3. - What are your experiences with the e-learning?
   - Negative, positive?
   - [For how long have you been using the e-learning platform?]
   - [How frequent do you use the e-learning platform?]

4. - How do you use the e-learning platform?
   - Do you think there is need to train students in using the ML e-learning platform? [Which kind of training?]
   - What kind of information should be available to ML students to make best use of e-learning platform? How should the information be communicated?

5. How does the e-learning contribute to your studies?
   - What kind of e-learning materials are most useful (least useful) to you and why?
   - How does the e-learning platform contribute to your medical practice?

6. Do you think the current content reflects the needs of the ML curriculum? [Why yes or why not?] [What is missing from your perspective?]

7. Do you think you will use the e-learning platform after graduation?
   - How would you use the e-learning platform after graduation?
   - How could the ML e-learning platform be useful to ML practitioners?

Students understanding, experiences and views on the current e-learning tablet

8. What do you think about the tablet?
   - What are your experiences with the tablets? [Positive, negative, what are important aspects to you of the tablet?] Do you think the tablet is well suited for ML students?

9. How do you use the tablet?
   - Do you think there is need to train students in using the tablet? [Which kind of

training?]

10. Do you access e-learning contents on the tablet? How do you access the ML e-learning on the tablet? How were your experiences in using the Moodle Mobile?

11. Have you experienced challenges with the tablet? What kind of challenges? Have the challenges been addressed? [Could the challenges with the tablet be addressed better?]

12. If you happen to encounter a problem with the tablet, which channel of communication would you use?

13. Do you think the tablet is well-suited for ML practice?

14. Do you think the tablet helps you in accessing medical information? How do you get information?
    - How do you think the tablet contributes to your health care practice?

Challenges of students using the e-learning platform

15. Do you prefer using the website or the tablet? Why?

16. What challenges can you identify for the ML e-learning platform and the tablets at CCHS? How can these challenges be addressed?

Recommendations of students for the e-learning platform

17. What recommendations would you give to the current e-learning platform and tablet? - - What could help you (with regards to the ML e-learning) to become a better MLP? How should the ML e-learning look like in 5 years? [How should it develop?]

18. Do you think the lecturers are using the e-learning platform effectively?
    - From your perspective, how should lecturers make use of the platform to make it a good learning tool for the ML program?
    - How would lecturers ideally employ or support the ML e-learning platform?

19. Is there anything that you want to add to this discussion?

⇒ General Probes for the Focus Group Interview:
- Can you talk about that more?
- Help me understand what you mean.
- Can you give an example?
- Thank you. What do other people think?

- Let’s have some other comments.
- Would you explain that further?
- Would you give an example?

End of discussion

1. Summarize key points of the respondent.
2. Ask the interviewee if there is anything they would like to add.
3. Thank interviewee for their time.
