# Supplementary material for: Authorship Correction: Perspectives of Nonphysician Clinical Students and Medical Lecturers on Tablet-Based Health Care Practice Support for Medical Education in Zambia, Africa: Qualitative Study
Source: JMIR Mhealth Uhealth. 2019 Apr 3;7(4):e13431. doi: 10.2196/13431 (PMC6468332; doi:10.2196/13431)
Supplement: Multimedia Appendix 1 [file mhealth_v7i4e13431_app1.pdf]

HEIDELBERG
UNIVERSITY
HOSPITAL

Heidelberg University Hospital | Im Neuenheimer Feld 672 | 69120 Heidelberg

Sandra Barteit, M.A.

Head of IT, Researcher
Heidelberg Institute of Global Health

Corrigendum

JMIR mHealth and uHealth
Editors

17th January, 2019

Corrigendum for published manuscript (#12637): "Perspectives of Nonphysician Clinical Students and Medical Lecturers on Tablet-Based Health Care Practice Support for Medical Education in Zambia, Africa: Qualitative Study"

Dear Editors

We have proofread the manuscript with the title **"Perspectives of Nonphysician Clinical Students and Medical Lecturers on Tablet-Based Health Care Practice Support for Medical Education in Zambia, Africa: Qualitative Study"** quite meticulously before giving the final approval for publication in JMIR mHealth and uHealth. However, we have found that - unfortunately - one author was not part of the co-authors of this publication (Annel Bowa), although initially he has been a co-author and he also signed the *Publication Form for JMIR Publications Authors*, which was uploaded for this publication on the JMIR platform.

Therefore, we would like to ask you to correct the published manuscript and add the co-author Annel Bowa to the currently listed co-authors.

The corrected list of authors should read as follows
(the correction is highlighted in bold):

Sandra Barteit[1], MA; Florian Neuhann[1], MD; Till Bärnighausen[1,2,3], MSc, MD, ScD, PhD; **Annel Bowa[5], MSc;** Sigrid Lüders[4], MD; Gregory Malunga[5], MSc; Geoffrey Chileshe[5], BSc; Clemence Marimo[6], MD; Albrecht Jahn1, MSc, MD, PhD

Thank you for your understanding.

All authors approve of the adding of the co-author Annel Bowa:

Sandra Barteit | Florian Neuhann | Till Bärnighausen

Annel Bowa | Sigrid Lüders | Gregory Malunga

Geoffrey Chileshe | Clemence Marimo | Albrecht Jahn

Im Neuenheimer Feld 130.3
69120 Heidelberg
Fon +49 6221 56-34030
Fax +49 6221 56-5948
barteit@uni-heidelberg.de
www.klinikum.uni-heidelberg.de

UK
HD

With best regards,

Sandra Barteit
(corresponding author)
